# Consumption and biochemical impact of commercially available plant-derived nutritional supplements. An observational pilot-study on recreational athletes

**DOI:** 10.1186/1550-2783-9-28

**Published:** 2012-06-19

**Authors:** Paolo Borrione, Marta Rizzo, Federico Quaranta, Emanuela Ciminelli, Federica Fagnani, Attilio Parisi, Fabio Pigozzi

**Affiliations:** 1 Department of Health Sciences, University of Rome “Foro Italico”, Piazza Lauro de Bosis 15, 00194, Rome, Italy

**Keywords:** Nutritional supplements, Ergogenic, Ecdysteroids, Phytoestrogens, Vegetal sterols, Athletes

## Abstract

**Background:**

A growing consumption of natural (plant-derived) dietary supplements with ergogenic aims, with particular regard for *ecdysteroids*, *phytoestrogen*s and *vegetal sterols*, has been registered over the last years among “recreational” athletes. The present study was carried out in order to evaluate the real knowledge of plant-derived nutritional supplements among physically active people as well as their real consumption. Additional aim was to evaluate the effects of these supplements on the health profile of the users.

**Methods:**

Twenty-three trained subjects who habitually used natural dietary supplements, and 30 matched controls were analyzed for plasma biochemical markers and hormonal profile.

**Results:**

The laboratory tests revealed the absence of any sign of organ toxicity/damage in both athletes and controls. On the contrary, hormone profiles revealed marked alterations in 15 (65%) out of the 23 of investigated athletes. Specifically, 10 males presented increased plasma levels of progesterone, 15 subjects presented abnormal estrogen levels, including 5 (2 F and 3 M) presenting a “dramatic” increased estrogen values and 2 two males with increased estrogen levels, increased testosterone levels and associated suppression of luteinizing hormone and follicle-stimulating hormone.

**Conclusions:**

The results of the present study highlighted that the habitual consumption of plant-derived nutritional supplements is frequently associated with significant hormonal alterations both in male and female subjects. Although these biochemical alterations were not associated with signs or symptoms of organ toxicity/damage at the moment of the study, it cannot be excluded that, in the mid/long-term, these subjects would suffer of health problems secondary to chronic exposure to heavily altered hormonal levels. Further large scale studies are needed to confirm the results of this pilot study as well as to investigate the biological mechanisms at the base of the observed hormonal alterations.

## Background

It is commonly accepted that nutritional habits play an important role in the individual capacity of reaching optimal physical performance and this idea has been strongly underlined by the American Dietetic Association [1]. Unfortunately, a parallel nutritional information pathway is growing day by day promoting innovative diets able, in theory, to enhance physical performances. Usually, the information provided to the public is to combine, to a defined nutritional regimen, specific supplements with the aim of reducing the length of time needed for reaching the desired results. Nowadays, the culture of dietary supplementation is widely diffused not only among professional athletes, but also among “recreational” athletes as well as active subjects. Indeed, the global supplement use in athletes is estimated ranging between 40% and 88% [2], showing an increasing diffusion among adolescents [3]. Common supplements used with ergogenic intents include: creatine, proteins, carbohydrates, aminoacids, vitamin complex and caffeine [4]. However, beside these “traditional” supplements, a growing consumption of natural (plant-derived) products has been registered over the last years. It is estimated that more than 1400 herbs are commonly commercialized for medicinal uses worldwide and these supplements represent a multi-billion-dollar business. In the sport environment, these products are usually marketed as performance enhancing aids and they are presented as legal and free of side effects, according to the misconception that “natural” corresponds to “not harmful”. However, the publicized effects of these products and the recommended dosages are often based on little or no scientific evidence, leading the scientific community to a great concerns when considering their safety [5].

Unfortunately, the sport environment has shown an increasing interest in those “alternative natural approaches”. In fact, it is appealing for the athletes the use of a natural therapeutic variant which promises similar activity to the pharmacological approach, in term of increasing physical performances, which is not considered doping and which is considered side-effect free. In this setting, herb derived products are usually suggested because the high title of active principles promises results similar to those obtained with pharmaceutical drugs but in absence of side effects and without the risk of testing positive for doping. Among the “natural” supplements, the most “attractive” are those containing plant-derived hormones such as *ecdysteroids*, *phytoestrogen*s and *vegetal sterols* and other substances with referred hormone modulating properties such as *tribulus terrestris*.

Ecdysteroids are the steroid hormones of arthropods. They also occur in certain plant species, where they are known as phytoecdysteroids and are believed to contribute to the deterrence of invertebrate predators. In insects, they regulate moulting and metamorphosis and have been implicated in the regulation of reproduction and diapause. Most actions of ecdysteroids are mediated by intracellular receptor complexes, which regulate gene expression in a tissue and development specific manner. Ecdysteroids are apparently non-toxic to mammals and a wide range of beneficial pharmacological (adaptogenic, anabolic, anti-diabetic, hepatoprotective, immunoprotective, wound-healing, and perhaps even anti-tumour) activities are claimed for them [6]. Moreover, the reported anabolic properties have led to a large (and unregulated) market for ecdysteroid-containing preparations, the most of which are advertised on specialized websites as legally allowed and non-toxic substances useful to gain muscular mass [7].

Phytoestrogens have acquired popularity for a multitude of health benefits, including a lowered risk of osteoporosis, heart disease, breast cancer, and menopausal symptoms, that have been attributed to them. Consequently, a global movement towards increased consumption of phytoestrogen-rich foods and tabletized concentrated isoflavone extracts have been heavily promoted in western countries over the last two decades. However, more recently, phytoestrogens have been considered endocrine disruptors having the potential to cause adverse health effects [8], as well the effects of phytoestrogens in preventing osteoporosis and menopausal symptoms have not been confirmed in more recent studies [9-11].

Phytosterols (including campesterol, stigmasterol and sitosterol) are plant steroids with a similar chemical structure and cellular function to human cholesterol. They are recommended as dietary modifiers of serum lipids [12]. In addition, plant sterols exert beneficial effects on other lipid variables, such as apolipoprotein (apo) B/apoAI ratio and, in some studies, high-density lipoprotein cholesterol (HDL-C) and triglycerides [13] and may also affect inflammatory markers, coagulation parameters, as well as platelet and endothelial function.

Finally, the main claimed effect of Tribulus Terrestris (TT) is an increase of testosterone, anabolic and androgenic action, through the activation of endogenous testosterone production [4]. Even if this biological pathway is not entirely proven, TT is regularly used by many athletes as “legal” anabolic aid. However, different studies concluded that TT do not produce the large gains in strength or lean muscle mass that many manufacturers claim can be experienced within 5–28 days and the possible health risks deriving from TT assumption have not been investigated [14].

Most of the previously mentioned commercially available supplements have not been studied for long-term safety and it’s likely that many habitually users are not aware of the real efficacy of these products, or the adverse effects related to their consumption. Questions regarding their possible side effects on endocrine and reproductive systems should be raised even in light of their advertised high-dose use.

With those premises, the present study was carried out in order to evaluate the real knowledge of plant-derived nutritional supplements among physically active people, in order to quantify the real use of these supplements and to evaluate the effects of these supplements on the health profile of the users.

## Methods

### Study protocol

This observational pilot study was designed in agreement with the Declaration of Helsinki and approved by the local Ethical Committee. All subjects volunteered to the study and gave their informed consent.

The enrolled subjects were asked to fill out an anonymous questionnaire in order to obtain information about their knowledge and/or personal experience with plant-derived nutritional supplements. Those who declared to consume any of these products were included in the study as “users” who were asked to provide a blood sample for laboratory analysis.

### Subjects

Over a period of 6 months, 740 trained subjects (420 body builders, 70 cyclists, and 250 fitness athletes) were enrolled in the study.

All subjects have been training regularly for at least 1 year, 1–2 hours per day, 3–6 days per week and most of them had practiced the same, or other, sports in the past. All subjects, through the compilation of the anonymous questionnaire, denied the consumption of any prohibited substances.

Athletes were instructed to abstain from caffeine, alcohol and drug consumption and to refrain from any strenuous physical activity for 24 hours before the examination that consisted of a blood sampling in the morning (08:00 h, after an overnight fast) and a medical evaluation which included a detailed familiar, medical and sportive personal history and a complete physical examination.

### Laboratory analysis

**Figure 1:**
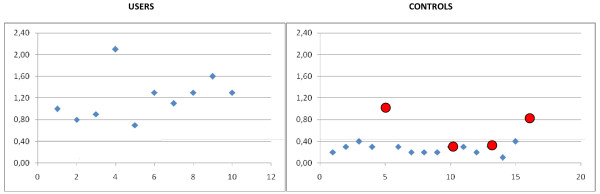
**Specific values of plasma progesterone in 10 “users”.** 0,4 ng/ml (red line) represents the upper limit of the reference range in males. Female subjects are indicated with red circles. The x axis represents the subject identification number and the y axis represents the values of plasma progesterone.

**Figure 2:**
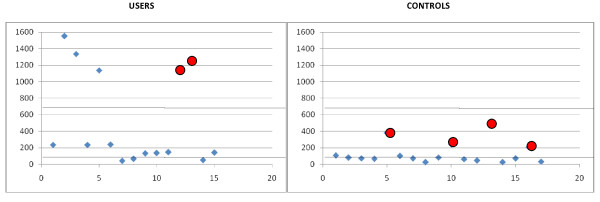
**Specific values of plasma estrogens in 15 users 13 males and 2 females (indicated with red circles).** 35 pg/ml represents the upper limit of the reference range in males (green lines), 650 pg/ml represents the upper limit of the reference range in females (red line). The x axis represents the subject identification number and the y axis represents the values of plasma estrogens.

Of the 740 athletes who completed the questionnaire, 26 declared to use plant-derived supplements and 23 of them gave their consent for the blood sample collection. Subjects n° 1, 3, 4 and 9 in Figure 1 and subjects n° 2, 3 and 5 in Figure 2 consumed, for at least 6 months 1,5 gr/die of a commercially available product containing Caffein, Citrus A., Zingiber, Guggul, Cacao, Naringina and Bioperine. Subjects n° 2, 5,and 6 in Figure 1 and subjects 1, 4, 9 and 12 in Figure 2 consumed, for at least 1 year, 3 gr/die of a commercially available product: 5-Methyl-7Methoxyisoflavone, 7-Isopropoxyisoflavone, 20-Hydroxyecdysone, Secretagogues, Triboxybol, Saw Palmetto extract, Beta Sitosterol, Pygeum extract, Guarana extract and Cordyceps extract. Subjects n° 7 and 8 in Figure 1 and subjects n° 6 and 8 in Figure 2 consumed, for at least 1 year and at different dosages, a commercially available product containing Rhaponticum Carthamoides extract (in 1 case, subject 6 in Figure 2, associated with another commercially available product containing Ajuga Turkestanica and Rhaponticum Carthamoides root extract). The remaining subjects consumed high doses of soy derived proteins (2–2.5 gr/kg/die for at least 1 year in some cases associated with Muira Puama and/or Gotu Kola extracts). All subjects also consumed daily different proportions of vitamins, proteins and branched-chain amino acids.

In addition, 30 subjects matched for age, gender, sport discipline, body mass index (BMI) and training volume were recruited as controls among those who denied the use of any nutritional supplements were enrolled as controls.

Blood samples were collected in SST II tubes with serum separator gel, immediately frozen and analyzed within the same day. Testosterone, Dehydroepiandrosterone (DHEA), Estrogens, Progesterone, luteinizing hormone (LH), follicle-stimulating hormone (FSH), thyroid-stimulating hormone (TSH), FT3, FT4 and Cortisol were analyzed by the immunometric method (Axym abbott Diagnostic Laboratories, Abbott Park, Illinois, USA). Urea, creatinine, aspartate aminotransferase (GOT), alanine aminotransferase GPT), lactate dehydrogenase (LDH), creatine kinase (CK), gamma glutamyl transpeptidase (GGT), alkaline phosphatise (APH), total and partial bilirubin, were measured spectrophotometrically by clinical-chemistry analyzer Integra 800 (Roche).

### GC/MS analysis

Since androgenic anabolic hormones have been frequently detected in steroid-free nutritional supplements [15,16], three natural products, used by 20 out of the 23 users, (Animal Stuck, EnerZona soy protein and TestostroGrow) were analyzed at the World Anti-Doping Agency (WADA) accredited Laboratory of Rome, following the appropriate authorization, to test their eventual contamination by steroid hormones for a total of 20 different hormones.

Sample Preparation: 1 g of powder was dissolved in carbonate buffer (PH:9), 50μL of internal standard (17 α-methyl-testosterone, final concentration 500 ng/mL) were added and the extraction was performed with 10 mL of pentane in a multimixer for 5 minutes. The organic layer was separated and evaporated under nitrogen at 70 °C. The dry residue was derivatized using 50μL of TMSJ at 75° C for 30 minutes. 2 μL of the derivatized layer were injected into a gas cromatograph connected to a mass spectrometer.

Instrumental Conditions: GC/MS was performed on an HP 6890 mass selective detector (Agilent Technologies, Tokio, Japan) connected with a 5973 quadruple mass spectrometry, with ionization energy modality, at 70 eV and SIM acquisition. The fused-silica capillary column used was HP1 with 0.20 mm diameter and 0.11 μm film thickness). Helium was used as a carrier gas (flow rate: 1 mL/min, split ratio 1:10).

### Statistical analysis

Database management and all statistical analyses were performed using the Statistica 6 for Windows software package (Statsoft Inc., Tulsa, OK). Normality of data was assessed by the Wilk-Shapiro’s test. Differences were analysed by means of the two-tailed Student’s *t* test. If a significant difference was present, a Dunn’s post hoc test was used to locate the difference. Levels of statistical significance were set to p < 0.05.

## Results

### Knowledge and use of nutritional supplements

Overall, plant-derived nutritional supplements resulted poorly known among the 740 enrolled subjects. Indeed, 45% of them declared not knowing any of te substances in the list. 24% of them declared knowing only phytoestrogens, 26% only vegetal sterols and only 5% declared knowing ecdysteroids. Overall, the use of these substances resulted extremely limited among the enrolled subjects (3%).

### Health status

The laboratory tests revealed the absence of any sign of organ toxicity/damage in all the subjects enrolled as shown in Table 1. Similarly, no significant differences between users and controls were found when considering the value of cortisol, LH, FSH, TSH, FT3, FT4 (Table 2). On the contrary, sex hormone profiles revealed marked alterations in 15 (65%) out of the 23 of investigated athletes, while no alterations were found in the control group (Table 2). Specifically, ten male subjects presented increased plasma levels of progesterone (Figure 1). Fifteen subjects presented abnormal estrogen levels, including 5 subjects (2 female and 3 males) presenting a “dramatic” increased estrogen values (Figure 2). Finally, two male subjects with increased estrogen levels (subjects 11 and 15 in Figure 2) presented concomitant increased testosterone levels associated with suppressed LH and FSH.

**Table 1:** Biochemical values in users and controls

	**USERS**	**CONTROLS**	**REFERENCE RANGE**	**P VALUE**
GOT (U/l)	24,1 ± 7,7	22,4 ± 6,4	5 - 40	NS
GPT (U/l)	22,9 ± 10,3	24 ± 12,8	10 - 40	NS
GGT (mU/ml)	23,1 ± 11,9	19 ± 13	7 - 85	NS
APH (U/l)	60,1 ± 16,7	59,9 ± 17,4	50 - 136	NS
LDH (U/l)	172 ± 38,5	161,1 ± 40,1	120 - 250	NS
Total Bilirubin (mg/dl)	0,9 ± 0,5	0,69 ± 0,46	0,2 - 1,3	NS

**Table 2:** Plasma hormone levels in users and controls

	**USERS**	**CONTROLS**	**REFERENCE RANGE**	**P VALUE**
LH (mlU/ml)	4,3 ± 3,3	4,9 ± 5,5	0 - 25	NS
FSH (mlU/ml)	3,7 ± 2,3	3,9 ± 3,9	0 - 25	NS
PROGESTERON (ng/ml)	0,8 ± 0,5	0,2 ± 0,9	0,1 - 0,4	0.006
TESTOSTERON (ng/dl)	539 ± 391	383,8 ± 187,6	250 - 850	NS
ESTROGENS (pg/ml)	363 ± 508,7	21,8 ± 33,5	15 - 35	0.000
DHEA (ng/ml)	2,8 ± 1,9	5,3 ± 2,4	1 - 7,5	0.000
FT3 (pg/ml)	3,2 ± 0,5	3,4 ± 0,5	1,5 - 4,5	NS
FT4 (ng/ml)	1,4 ± 0,5	1 ± 0,1	0,75 - 1,95	NS
TSH (micrU/ml)	1,5 ± 0,6	1,32 ± 0,8	0,5 - 4	NS
CORTISOL (mcg/dl)	14 ± 3,6	13,3 ± 5	4 - 20	NS

All of the subjects presenting hormone alterations were submitted to an additional complete clinical evaluation which revealed the absence of any disease or pathological conditions. In particular, no alteration of the secondary sexual characters were found (particularly notable the absence of gynecomastia in men with elevated progesterone levels). However, as a form of “good medical practice” all these subjects were advised to stop the consumption of potentially unsafe products and were recommended for a careful medical follow-up.

### Dietary habits

All the users who presented with abnormal sexual hormone levels declared of regularly consuming multiple dietary supplements, including “traditional” and “natural” compounds. Interestingly, those with abnormal estrogen levels shared the consumption of high dosage of soy protein (2 gr/Kg/die).

Subjects with abnormal estrogen levels associated with increased progesterone levels consumed products containing ecdysteroids. Finally, those with increased testosterone levels consumed both high dosage of soy protein and products containing ecdysteroids and tribulus terrestris.

### GC/MS analysis of the commercially available products

The GC/MS analysis excluded the contamination of the texted products by steroid hormones.

## Discussion

As far as our knowledge goes, this is the first study investigating the real consumption of plant-derived nutritional supplements with ergogenic intent on recreational athletes and the possible side effects deriving from this practice. The study highlighted that, among Italian athletes, these products are poorly known when compared to the “traditional” supplements and that their use is still limited. Noteworthy, even with the limitations due to the smallness of the sample, the study seems to demonstrate that the regular use of this types of nutritional supplements, even in the form of poly consumption, do not cause organ suffering or damage, in particular to liver and kidneys. On the contrary, the significant alterations of the sexual hormone profile, emerged in habitual users, represents the major finding of this investigation.

The use of nutritional supplements with ergogenic aims is as ancient as the sport itself [17] and it’s almost utopian thinking that sportive practice would be completely free from these substances. Indeed, athletes are mainly vulnerable to substance use, and abuse, in situations where much depends on sporting success; however, the use of ergogenic supplements is currently an accepted practice also among the “recreational” athletes and such practice is favored by an aggressive market, mainly expressed trough dedicated websites. Actually, it has been estimated that over 30 thousand different products referred to as “nutritional supplements” are commercially available [18]. Over the last years, this business have been considerably enlarging for the introduction of the so called “natural” or herbal products including those with hormonal effects (ecdysteroids, phytoestrogens, phytosterols and *tribulus terrestris*). These products have been quickly spreading all over the western world mainly through the network, even though they still remain less known in respect to the traditional supplements, as our investigation highlighted.

Undoubtedly, “natural” products are more appealing than the chemical ones because of the common misconception that what is natural is also harmless. Actually, many athletes trust in these products that promise effects comparable to those of steroids hormones or growth hormone, without the side effects of those prohibited substances. However, most of the users do not know that the ergogenic gains advertised for most of the nutritional supplements, including the natural ones, are often not based on scientific evidence and the possible risks for health deriving from their mid-term and long-term consumption are still not known.

The notable finding of this study is the evidence of highly significant alteration of sexual hormone levels in habitual users of plant-derived nutritional supplements. Although these biochemical alterations were not associated with signs or symptoms of disease at the moment of the study, it cannot be excluded that, in the mid/long-term, these subjects would suffer of health problems secondary to chronic exposure to heavily altered hormonal levels.

Mechanisms at the basis of these alterations are not known and the exact consequences are not predictable. However, it is known that hyperestrogenism may cause significant medical problems in both males and females. In particular, hyperestrogenism has been related to gynecomastia, hypogonadism, reduced fertility in men, macromastia, enlarged uterus and menstrual irregularities in women [19]. In addition, hyperestrogenism represents a major risk factor for the rare male breast cancer [20,21].

In our study, hyperestrogenism was observed in athletes who consumed high dosage of soy protein, the main food source of phytoestrogens. Actually, besides the known beneficial effects, these herbal hormones may have possible unfavorable effects in humans by interfering with the function of normal cellular activities, such as receptor-mediated signal transduction and DNA replication, as well as genotoxic, mutagenic and proliferation of some cancer cells [22]. This observation must be carefully considered when reflecting upon the increasing number of vegan and vegetarian athletes for whom soy represents the main source of protein, consumed in the form of protein powders and bars [23-25].

## Conclusions

With the exception of soy protein, the knowledge and the use of plant-derived nutritional supplements, with ergogenic aims in recreational athletes, appears to be limited even though the flourishing market of these products on internet sites portray the contrary. Nonetheless, the results of the present study confirmed that “natural” does not necessarily mean harmless and safe, and strongly advises against the use of nutritional supplements for superficial purpose. Undoubtedly, further larger scale studies are needed to confirm the results of this pilot study as well as to investigate the biological mechanisms at the base of the observed hormonal alterations.

## Competing interests

The author(s) declare that they have no competing interests.

## Authors’ contributions

PB was responsible for the conception and design of the study as well as the preparation of the manuscript; MR contributed to the design of the study and the preparation of the manuscript; FQ, EC and FF were responsible for the patients recruitment and data collection; AP contributed to data collection and analysis; FP contributed to the study design, was responsible for the final approval of the manuscript. All authors read and approved the final manuscript.
